# Dietary Inflammatory Index Score and Its Association with Body Weight, Blood Pressure, Lipid Profile, and Leptin in Indonesian Adults

**DOI:** 10.3390/nu11010148

**Published:** 2019-01-11

**Authors:** Harry Freitag Luglio Muhammad, Marleen A. van Baak, Edwin C. Mariman, Dian Caturini Sulistyoningrum, Emy Huriyati, Yi Yi Lee, Wan Abdul Manan Wan Muda

**Affiliations:** 1 Department of Nutrition and Health, Universitas Gadjah Mada, Yogyakarta 55281, Indonesia; dian.csulis@gmail.com (D.C.S.); emy_huriyati@yahoo.com (E.H.); 2 NUTRIM School of Translational Nutrition and Metabolism, Department of Human Biology, Maastricht University, 6200 MD Maastricht, The Netherlands; m.vanbaak@maastrichtuniversity.nl (M.A.v.B.); e.mariman@maastrichtuniversity.nl (E.C.M.); 3 International Institute for Global Health, United Nations University, Kuala Lumpur 16150, Malaysia; leeyy.yiyi@gmail.com; 4 Center for Southeast Asian Studies, Kyoto University, Kyoto 606-8501, Japan; wanmanan@gmail.com

**Keywords:** dietary inflammatory index, obesity, triglyceride, HDL, leptin

## Abstract

It was previously reported that dietary intake is an important trigger for systemic inflammation and one of the lifestyle factors for the development of cardiovascular diseases. The aim of this study was to evaluate the association between Dietary Inflammatory Index (DII) score and body weight, blood pressure, lipid profile and leptin in an Indonesian population. This was a cross-sectional study conducted in 503 Indonesian adults. The DII score was calculated based on data of 30 nutrients and food components. Anthropometric profile, blood pressure, lipid profile, and leptin were measured. The association of these variables with the DII score was analyzed. The DII score was not associated with body weight, body mass index (BMI), body fat, waist circumference, hip circumference, systolic and diastolic blood pressure, triglycerides, and high-density lipoprotein (HDL) (both unadjusted and after adjustment for covariates). However, plasma leptin concentration was significantly associated with the DII score (B = 0.096, *p* = 0.020). Plasma leptin also increased significantly across tertiles of the DII score (ANCOVA, *p* = 0.031). This positive association between the DII score and plasma leptin concentration suggests a role for the inflammatory properties of the diet in regulating adipose tissue inflammation.

## 1. Introduction

Obesity affects millions of adults and children and this state of over-nutrition is responsible for an increasing economic and health burden worldwide [1,2]. Adiposity is associated with increased risk for non-communicable diseases (NCDs) such as type 2 diabetes mellitus, dyslipidemia, heart disease, and hypertension [1]. One of the mechanisms which may explain the interaction between obesity and the development of NCDs is systemic and adipose tissue inflammation [3,4]. The excess of adipose tissue induces increments in the production of leptin and pro-inflammatory cytokines as well as immune cell infiltration [4]. In addition, a reduction in anti-inflammatory immune cells in adipose tissue of individuals with obesity has been reported [5,6]. This combination leads to an inflammatory state in adipose tissue as well as the circulation of individuals with obesity [4].

Inflammation is also influenced by environmental factors (e.g., lifestyle, diet, physical activity) and genetic background [7]. It has been reported previously that the dietary pattern has an important role in affecting circulating inflammatory markers in adults [8]. Recently, a new tool has been developed to assess the inflammatory properties of the diet: the Dietary Inflammatory Index (DII) [9]. In this index, intake of nutrient and non-nutrient components of the diet are calculated and compiled into an index. The score on this index has been shown to be associated with systemic inflammation [10,11,12]. In the cross-sectional Spanish PREDIMED (*Prevención con Dieta Mediterránea*) study the DII score was associated with body weight and other anthropometric measures [13]. In the Spanish SUN (*Seguimiento Universidad de Navarra*) cohort, the DII score was associated with annual weight gain [14]. We have shown that the DII score was associated with weight regain following a weight loss program in Dutch overweight and obese individuals [15]. 

Inflammation is associated with the development of chronic diseases and is an important link between obesity and cardiovascular diseases [3,16]. It was previously suggested that inflammation plays an important role in the disturbance of blood pressure and lipid profile [3,16,17]. This notion is supported by several studies in western societies which showed that the inflammatory properties of the diet were associated with early predictors of cardiovascular diseases such as hypertension, and higher plasma triglyceride and lower high-density lipoprotein (HDL) cholesterol concentrations [18,19,20].

Currently, there is limited evidence on the application of the DII in Asian countries. To our knowledge, there is only one report from a small cross-sectional study conducted in female school teachers in Myanmar, which found no association between the DII score and overweight [21]. Evaluation of the DII in different population settings is important because dietary practices vary amongst cultures and might have an impact on the DII value. Additionally, because other factors such as environments, lifestyle, and genetic background are also different among population settings, this might influence the relationship between the DII score and metabolic health. Therefore, the aim of this study was to evaluate the associations between the DII score and body weight, blood pressure, lipid profile, and leptin in an Indonesian population.

## 2. Materials and Methods 

These analyses are part of an Indonesian cohort study that investigates the effect of lifestyle and genetic variation on metabolic syndrome in adults. It is a secondary cross-sectional analysis of baseline data of this study that was carried out among adult men and women between 19 and 56 years of age, living in the urban area of Yogyakarta, Indonesia. A total of 503 individuals, stratified for sex, were recruited from randomly selected neighborhoods from five sub-districts which were selected. 

The inclusion criteria were: permanent residence (at least 2 years) in the area and agreement to become subject of this study by signing the informed consent. The exclusion criteria were: diagnosis of chronic diseases such as diabetes, cardiovascular disease, or cancer, pregnancy at the time the study was conducted, current or prior cigarette smoker status, strict diet, and problems with walking or conducting physical activity in the prior 6 months. Subjects who consumed drugs to treat the clinical features of cardiovascular diseases (such as blood pressure-, lipid-, and/or glucose-lowering drugs) were also excluded from this study. Ethical clearance was obtained from the Medical and Health Research Ethics Committee (MHREC) Faculty of Medicine, Universitas Gadjah Mada, Indonesia (KE/FK/791/EC/2015). This study followed the ethical guidelines of the 1975 Declaration of Helsinki. 

Obesity status was defined by body mass index (BMI) and calculated by dividing body weight with the square of height. Body composition was defined by percent body fat. Body weight and body fat were measured using a digital body mass scale and bioelectrical impedance (0.01 kg precision, Omron Karada Scan HBF-375, Osaka, Japan). Height was measured using a wall-mounted tape measure (0.1 cm precision, GEA medical, Jakarta, Indonesia). Waist and hip circumference were measured using a non-elastic tape (0.1 cm precision). All anthropometric measurements were done by trained personnel using calibrated instruments. 

Blood pressure was measured using Omron HEM 7120 Automatic Blood Pressure (Omron, Japan). This measurement was done after at least 10 minutes rest from recent activity and the participants were asked to sit in a comfortable sitting position with their left arm fully exposed and resting on a supportive surface at the heart level. Blood pressures were measured on the left arm using appropriate cuff size.

Data on dietary intake were collected using a validated Semi-Quantitative Food Frequency Questionnaire (SQ-FFQ) and the analysis was based on Indonesian food database and United States Department of Agriculture [22]. Data of habitual consumption of food items that were collected using the SQ-FFQ were translated into daily intake [23]. Data collection for dietary intake was done by a face-to-face interview between trained nutritionists and subjects.

The Dietary inflammatory Index (DII^®^) score is a calculated parameter that gives an overall picture of the inflammatory properties of the diet. An individual’s diet is considered more pro-inflammatory when the DII score is more positive, while the diet is considered more anti-inflammatory when the DII score is more negative. The DII score was calculated according to Shivappa et al. [9]. The calculation of the dietary inflammatory index score was based on 30 nutrients and food components including total energy, protein, carbohydrate, total fat, saturated fat, trans fat, mono-unsaturated fatty acid (MUFA), poly-unsaturated fatty acid (PUFA), omega-3 fatty acid, omega-6 fatty acid, cholesterol, fibre, magnesium, iron, selenium, zinc, vitamin A, vitamin C, vitamin D, vitamin E, thiamin, riboflavin, vitamin B6, vitamin B12, folate, niacin, beta-carotene, alcohol, caffeine, and tea. No data were available for the other dietary factors included by Shivappa et al. [9] in their dietary inflammation index such as rosemary, oregano, pepper, turmeric, saffron, onion, ginger, garlic, and polyphenols (i.e., isoflavones and anthocyanidins). To derive the individual DII scores, the global average intake (taken from Shivappa et al. [9]) was subtracted from the reported daily intake of each nutrient in the FFQ and divided by the standard deviation of the global daily intake, rendering a z-score which was converted into a centered percentile score. This score was then multiplied with an overall inflammatory effect score. All 30 (out of 48) included individually calculated nutrient-specific effect scores were then summed to obtain the DII score.

Data on physical activity was collected using the International Physical Activity Questionnaire (IPAQ) [24]. This questionnaire contains information on the intensity and duration of several activities including work/job, transportation, house-related work and maintenance, recreation, exercise and leisure-time physical activity. Each activity has a unique metabolic equivalent of task (MET) score, which represents the amount of energy used for a certain type of activity. In order to obtain an overall picture of the individual’s physical activity, all the activities that have been reported in IPAQ are transformed into MET-minutes/week. The SQ-FFQ and IPAQ were developed, validated and used before [23,24,25].

From each participant a 10-mL blood sample was collected in ethylenediaminetetraacetic acid (EDTA)-containing tubes. After collection, blood plasma and buffy coat were separated by centrifugation. Plasma HDL cholesterol and triglyceride concentration were measured using the cholesterol oxidase phenol 4-aminoantipyrine peroxidase (CHOD-PAP) and glycerol phosphate oxidase (GPO) methods, respectively (Diasys, Holzheim, Germany). Plasma leptin concentration was measured using an enzyme-linked immunosorbent assay (DRG, Springfield Township, NJ, USA). 

Statistical analyses were conducted using JASP (University of Amsterdam, the Netherlands) [26]. The relationships between the DII score as independent variable and body weight, blood pressure, lipid profile and leptin concentration as dependent variables were analyzed by linear regression analysis (Model 0, unadjusted). Adjustments for age, sex, physical activity and energy intake, and BMI where relevant, were done. Model I represents a regression analysis with adjustment for age, sex, and BMI (for blood pressure, triglycerides, HDL and leptin). Model II represents a regression analysis with adjustment for age, sex, energy intake, physical activity and BMI (for blood pressure, triglycerides, HDL and leptin). In addition, we made groups based on tertiles of the DII score and compared the variables by ANOVA (analysis of variance) or ANCOVA (Analysis of covariance). The ANCOVA analysis adjusted for age, sex, energy intake, and physical activity when analyzing differences in anthropometric measures among DII score tertiles. For analysis of blood pressure, lipid profile, and leptin differences between DII score tertiles, ANCOVA adjusting for age, sex, body mass index, energy intake, and physical activity was used.

## 3. Results

Characteristics of study participants are shown in Table 1. In this study, 503 adults (men 50.1% and women 49.9%) living in Yogyakarta were investigated. Data on the characteristics of men and women separately can be found in Supplementary Table S1. 

Data of dietary intake of study participants are shown and compared with global data of dietary intake based on Shivappa et al. [9] (Table 2). Compared to global averages, participants in this study had a relatively high intake of pro-inflammatory components such as total energy, carbohydrate, iron, and vitamin B12. They had a relatively low intake of anti-inflammatory components such as omega 3 fatty acid, omega 6 fatty acid, niacin, vitamin A, vitamin D, vitamin E, alcohol, and caffeine, but a relatively high intake of other anti-inflammatory components such as fiber, magnesium, selenium, vitamin C, folic acid, and tea.

Data on the relationship between the DII score and anthropometric measures are shown in Table 3. We found that the DII score was not associated with any of the anthropometric measures (all *p* >0 .05). This remained after adjustment for age, sex, physical activity and energy intake (Table 3). The DII score was also not associated with systolic and diastolic blood pressure, triglycerides and HDL cholesterol (all *p* >0.05). These associations also remained non-significant after adjustment for covariates (Table 3). However, after correction for age, sex, BMI, energy intake, and physical activity we found that the DII score was positively associated with plasma leptin concentration (Table 3). The sex specific analyses of the relationship between the DII score and all measured parameters are shown in Supplementary Table S2. There were no sex-related differences in the relationships between the DII score and any of the measured parameters.

To further clarify the association between DII score, anthropometric measures, blood pressure, lipid profile and leptin, we divided subjects into three tertiles based on their DII score (Table 4). No significant differences among the 3 tertiles (ANOVA, all *p* >0.05) were found. However, after adjustment for age, sex, energy intake, phsyical activity and body mass index, the increase in leptin concentration across DII score tertiles was statistically significant (ANCOVA, *p* = 0.031). 

## 4. Discussion

This study was aimed to evaluate the relationship between DII score, body weight, blood pressure, lipid profile and leptin in Indonesian adults. We found that DII score was not correlated with any of the anthropometric measures, blood pressure or lipid profile. Interestingly, we showed that the DII was positively correlated with plasma leptin concentration after correction for age, sex and BMI, energy intake and physical activity.

We compared the intake of the components of the DII in our Indonesian population with the global averages described by Shivappa [9]. Participants in this study had higher intake of pro-inflammatory components such as total energy, carbohydrates, iron and vitamin B12 and a lower intake of anti-inflammatory components such as omega 3 fatty acids, omega 6 fatty acids, niacin, vitamin A, vitamin D, and vitamin E. The mean energy intake of men was 11,285 kJ/day and this matches the Indonesian dietary recommendation for male adults (11,406 kJ/day) [27]. The mean energy intake of women was 10,277 kJ/day and this was slightly higher than the Indonesian dietary recommendation for female adults (9418 kJ/day) [27]. Results from this study provide a practical implication that can help reduce the inflammatory properties of diet of individuals in the study population. This can be done by reducing the consumption of carbohydrate rich foods (e.g., rice, sugar and wheat-based products) and increasing consumption of unsaturated fat and protein rich foods (such as eggs and fatty fish). This is because, although protein is considered as pro-inflammatory nutrient, it has a lower inflammatory effect score than carbohydrate [9]. Additionally, unsaturated fats are anti-inflammatory nutrients and foods that are rich in unsaturated fats usually also contain fat soluble vitamins such as vitamin A, D, and E [22]. Those vitamins were reported to be lower in these study participants compared to the global dietary intake. 

This study was initiated by our earlier finding that in overweight/obese Caucasian men and women who undertook a weight loss program, the DII score was correlated with weight regain during follow-up [15]. In this study we showed that the DII score was not correlated with body weight in Indonesian adult men and women. The relationship between the DII score and obesity indices has also been evaluated elsewhere. In a cross-sectional analysis of the Spanish PREDIMED trial, the DII score was associated with BMI only in women but not in men [13]. In Myanmar, a small cross-sectional study among overweight and non-overweight female school teachers showed that the DII score was not associated with overweight [21]. A role for DII in the development of obesity was indicated by Ramallal et al. [14]. They showed that in a non-overweight adult Spanish cohort the DII score was not associated with BMI at baseline, but a higher DII score was associated with a higher body weight increment after 8 years of follow up and a higher risk of developing overweight or obesity. Based on these studies and our findings, the effect of the DII on body weight remains inconclusive. Well-controlled longer-term intervention studies are required to shed more light on the role of DII in body weight regulation. 

There is more convincing evidence that inflammation is an important link between obesity and its cardiovascular co-morbidities [3,4,5,6]. Previously it was suggested that low-grade inflammation was associated with the development of dyslipidemia [3]. In this study, we found that the DII score was not correlated with components of the lipid profile, such as triglycerides and HDL. In contrast, Neufcourt et al. showed in a large (*n* = 3 726) cohort of French adults [18] that at baseline, the DII score was positively associated with triglyceride level, but not with HDL cholesterol level. After a follow up of 13 years, the DII score was significantly associated with higher triglyceride and lower HDL cholesterol levels. The association between the DII score and lipid profile was also confirmed by a small scale study (*n* = 90) in Colombia [19]. These investigators showed that study participants with a higher pro-inflammatory diet (based on DII score calculation) had a significantly lower HDL level. Our intermediate size study in Indonesian adults could not confirm these findings. On the other hand, there was also no increased risk for low HDL cholesterol or elevated triglyceride concentrations with increasing DII score in a study in 447 U.S. police officers [28]. Dyslipidemia or disturbance of lipid profile including triglyceride and cholesterol levels have long been used as an early biomarker of cardiovascular diseases. However, this concept is now being challenged. Inflammation may be a more important biomarker of cardiovascular diseases [16]. This shift of paradigm is necessary because this might affect dietary approaches for cardiovascular diseases prevention. Instead of aiming to lower cholesterol and triglycerides, it might be more beneficial to lower the inflammatory properties of the diet as a means of prevention of cardiovascular diseases.

In this study we also showed that the DII score was not associated with blood pressure, neither in men nor women. This finding differs from studies previously reporting that the DII score was associated with increased risk for developing hypertension among middle-aged Australian women [20] and Polish adults [29]. On the other hand, Wirth et al. [28] also found no higher risk of elevated blood pressure with increasing DII score in U.S. police officers. 

We cannot exclude the possibility that the lack of association between the DII score and lipid profile and blood pressure is due to our inclusion criteria. We only included subjects without a clinical diagnosis cardiovascular diseases, diabetes, or treatment for dyslipidemia or high blood pressure, because we were interested in investigating the role of diet in the early onset development of chronic diseases and thus our population can be regarded as relatively healthy compared to the general Indonesian population.

In this study, we showed that the DII score was positively associated with plasma leptin concentration after adjustment for age, sex, BMI, energy intake, and physical activity. This finding was confirmed by analysis based on DII score tertiles, which showed that leptin concentration increased significantly across the tertiles. Leptin is produced by adipose tissue and its production increases with the progression of adiposity [30]. In the past few decades, there has been a growing interest in understanding the interaction between leptin, inflammation and oxidative stress. Leptin is a cytokine which is produced by adipocytes with an ability to induce inflammation. The pro-inflammatory properties of leptin have been suggested to be similar to those of immune cell-derived cytokines such as tumor necrosis factor alpha (TNF-α) and interleukin 6 (IL-6) [31,32]. In addition to inflammation, it was suggested that leptin plays an important role in the development of oxidative stress in obesity by inducing production of reactive intermediates such as H_2_O_2_ and hydroxyl radicals [33]. The accumulation of these processes can induce development of cardiovascular diseases. This is supported by several studies, which showed that a higher leptin concentration was associated with increased risk for cardiovascular diseases [34,35,36]. To our knowledge, only one other study has investigated the relationship between DII score and leptin [28]. In this cross-sectional study in U.S. police officers, the DII score was not associated with leptin concentration. 

There are several strengths and limitations in this study. The strengths of this study are the relatively large sample size and the non-Western population. However, there are several limitations to this study. First, because data collection was done using a SQ FFQ for Indonesian food consumption with limited data on bioactive components of foods, we cannot provide data on the intake of a number of herbs/ seasonings (such as oregano, rosemary, pepper, saffron, garlic, onion, and ginger) as well as polyphenols (such as flavonol, anthocyanidins, and eugenol), which are included in the original list provided by Shivappa et al. [9]. Additionally, there are some food components that have no known value for different types of PUFAs in the food databases used, which might have an impact on total omega 3 and 6 consumption. These might influence the relationships between DII score and outcomes in comparison to the full DII according to Shivappa et al. Second, we reported data on dietary intake amongst individuals who live in urban areas. Therefore, data from this study may not represent the intake of those that live in rural areas. Third, because of the design of this study, no conclusion about causality can be drawn. Fourth, measurements were performed at random times during the day and in the non-fasting state, which may have interfered with the potential association between the DII score and the outcome variables by increasing the variation. However, it was previously shown that in large cohorts the influence of normal food intake on changes in lipids, lipoproteins, and apolipoproteins is small and that the random non-fasting lipid profile remains a good predictor for cardiovascular diseases in humans [37]. However, leptin production has a diurnal variation [38] and is affected by fasting status [39], and therefore confirmation of our findings under better standardized conditions is needed. 

## 5. Conclusions

In summary, we showed that the DII score was positively correlated with the plasma leptin concentration, which is one of the markers of adipose tissue inflammation. This might explain the connection between the DII score and increased systemic inflammation as well as cardiovascular diseases in other studies. Because of the potential importance of diet in the development of inflammation, intervention studies that investigate the effect of manipulation of the inflammatory properties of the diet on the inflammatory process are warranted.

## Figures and Tables

**Table 1:** Characteristics of study participants.

Characteristics	All (*n* = 503)
Age (years)	41.6 ± 10.2
Anthropometric measures	
Body weight (kg)	62.6 ± 13.1
Height (cm)	158.5 ± 9.0
Body mass index (kg/m^2^)	24.9 ± 5.0
Body fat (%)	27.5 ± 8.7
Waist circumference (cm)	86.2 ± 12.7
Hip circumference (cm)	93.9 ± 11.2
Waist-to-hip ratio	0.92 ± 0.07
Blood pressure	
Systolic (mmHg)	128.6 ± 25.2
Diastolic (mmHg)	79.7 ± 14.8
Metabolic profile	
Triglycerides (mmol/L)	1.57 ± 0.82
HDL cholesterol (mmol/L)	1.18 ± 0.47
Leptin (ng/mL)	7.3 ± 8.8
Physical activity (METs-min/week)	5781 ± 5932
Dietary intake	
Energy (kJ/day)	10 838 ± 4 789
Protein (energy %)	12.5 ± 3.4
Fat (energy %)	23.1 ± 10.6
Carbohydrate (energy %)	64.3 ± 10.8
Dietary inflammatory index score	1.01 ± 7.29
% overweight ^a^	45.9
% obese ^b^	14.9

Values are presented as mean ± standard deviation. ^a^ body mass index ≥25 kg/m^2^ or higher, ^b^ body mass index ≥30 kg/m^2^ or higher, ^a,b^ percent of total number of study participants. HDL = high-density lipoprotein; MET = metabolic equivalent of task.

**Table 2:** Comparison of global dietary intake and dietary intake of Indonesian adults.

Intake	Global Dietary Intake	Dietary Intake of Study Participants
Pro-inflammatory components		
Carbohydrate (g)	272.2 ± 40.0	416.9 ± 202.8
Cholesterol (mg)	279.4 ± 51.2	177.8 ± 161.8
Energy (kj)	8 606 ± 1 414	10 838 ± 4 789
Iron (mg)	13.35 ± 3.71	18.1 ± 9.8
Protein (g)	79.4 ± 13.9	80.8 ± 41.9
Saturated fat (g)	28.6 ± 8.0	25.7 ± 19.6
Total fat (g)	71.4 ± 19.4	66.6 ± 45.9
Trans fatty acid (g)	3.15 ± 3.75	2.82 ± 4.72
Vitamin B_12_ (µg)	5.15 ± 2.70	7.38 ± 9.54
Anti-inflammatory components		
Alcohol (g)	19.98 ± 3.72	0.03 ± 0.07
Beta-carotene (µg)	3 718 ± 1 720	3 696 ± 3 721
Caffeine (g)	8.05 ± 6.67	0.09 ± 0.10
Fiber (g)	18.8 ± 4.0	24.3 ± 13.5
Folic acid (µg)	273.0 ± 70.7	403.0 ± 339.4
Magnesium (mg)	310.1 ± 139.4	392.1 ± 278.2
MUFA (g)	27.0 ± 6.1	29.5 ± 23.1
Niacin (mg)	25.9 ± 11.8	19.57 ± 9.14
Omega 3 fatty acids (g)	1.06 ± 1.06	0.46 ± 0.47
Omega 6 fatty acids (g)	10.80 ± 7.50	0.86 ± 0.80
PUFA (g)	13.88 ± 3.76	13.40 ± 9.64
Riboflavin (mg)	1.70 ± 0.79	1.97 ± 1.72
Selenium (µg)	67.0 ± 25.1	103.0 ± 49.6
Black/green tea (g)	1.69 ± 1.53	2.36 ± 2.60
Thiamin (mg)	1.70 ± 0.66	1.39 ± 0.71
Vitamin A (RE)	983.9 ± 518.6	574.3 ± 508.0
Vitamin B_6_ (mg)	1.47 ± 0.74	4.59 ± 3.71
Vitamin C (mg)	118.2 ± 43.5	152.3 ± 145.5
Vitamin D (µg)	6.26 ± 2.21	3.44 ± 5.88
Vitamin E (mg)	8.73 ± 1.49	2.74 ± 2.65
Zinc (mg)	9.84 ± 2.19	9.59 ± 4.75

MUFA = mono-unsaturated fatty acid, PUFA = poly-unsaturated fatty acid, RE = retinol equivalent.

**Table 3:** Standardized regression coefficients (B) and their standard error (SE) and *p*-value of the association between DII score and anthropometric variables, blood pressure, lipid profile, and leptin concentration.

Variables	Model 0 ^a^			Model I ^b^			Model II ^c^		
	B ^a^	SE ^a^	*p* ^a^	B ^b^	SE ^b^	*p* ^b^	B ^c^	SE ^c^	*p* ^c^
Body weight (kg)	−0.010	0.081	0.900	−0.011	0.078	0.801	−0.039	0.096	0.470 ^
BMI (kg/m^2^)	−0.012	0.031	0.693	−0.020	0.030	0.648	−0.080	0.036	0.132 ^
Body fat (%)	−0.018	0.054	0.736	−0.009	0.035	0.762	−0.044	0.043	0.222 ^
Waist circumference (cm)	−0.079	0.078	0.311	−0.048	0.074	0.251	−0.046	0.091	0.379 ^
Hip circumference (cm)	0.012	0.069	0.857	0.006	0.064	0.878	−0.049	0.079	0.347 ^
Systolic BP (mmHg)	0.221	0.154	0.153	0.066	0.134	0.088	0.034	0.165	0.481 *
Diastolic BP (mmHg)	0.128	0.091	0.159	0.065	0.082	0.107	0.046	0.102	0.355 *
Triglycerides (mmol/L)	−0.004	0.005	0.407	−0.037	0.005	0.388	0.045	0.006	0.406 *
HDL cholesterol (mmol/L)	0.0003	0.003	0.925	0.010	0.003	0.825	−0.046	0.004	0.400 *
Leptin (ng/mL)	0.101	0.061	0.102	0.105	0.042	0.002	0.096	0.052	0.020 *

^a^ Model 0, linear regression analysis without adjustment; ^b^ Model I, linear regression analysis with adjustment for age and sex; ^c^ Model II, linear regression analysis with correction for age, sex, energy intake, physical activity and BMI * or without BMI ^; BMI = body mass index; BP = blood pressure; SE = standard error; DII = Dietary Inflammatory Index.

**Table 4:** The differences on anthropometric measures, lipid profile, blood pressure and leptin among DII score tertiles.

Variables	DII Score Tertile 1 (<−1.0) (*n* = 169)	DII Score Tertile 2 (1.0–5.1) (*n* = 168)	DII Score Tertile 3 (>5.1) (*n* = 164)	*p*_ANOVA_ *	*p*_ANCOVA_
Anthropometric measures					
Body weight (kg)	61.9 ± 13.9	63.5 ± 11.0	62.3 ± 14.2	0.538	0.840 ^
Body mass index (kg/m^2^)	24.7 ± 4.8	25.3 ± 4.7	24.9 ± 5.5	0.523	0.470 ^
Body fat (%)	27.4 ± 8.6	27.5 ± 8.7	27.7 ± 9.0	0.937	0.543 ^
Waist circumference (cm)	86.2 ± 12.7	87.1 ± 12.4	85.4 ± 13.2	0.467	0.395 ^
Hip circumference (cm)	93.1 ± 10.3	95.0 ± 10.7	93.7 ± 12.5	0.301	0.209 ^
Systolic blood pressure (mmHg)	126.9 ± 25.2	129.3 ± 24.5	129.5 ± 25.9	0.590	0.847 ^#^
Diastolic blood pressure (mmHg)	79.1 ± 14.3	80.2 ± 14.4	79.9 ± 15.8	0.773	0.790 ^#^
Triglycerides (mmol/L)	1.6 ± 0.9	1.6 ± 0.8	1.5 ± 0.8	0.479	0.413 ^#^
HDL cholesterol (mmol/L)	1.2 ± 0.5	1.1 ± 0.4	1.2 ± 0.5	0.378	0.572 ^#^
Leptin (ng/mL)	6.2 ± 6.9	7.1 ± 8.5	8.6 ± 10.7	0.071	0.031 ^#^

* ANOVA analysis across DII score tertiles; ^ ANCOVA analysis across DII score tertiles with adjustment for age, sex, energy intake and physical activity; ^#^ ANCOVA analysis across DII score tertiles with adjustment for age, sex, body mass index, energy intake, and physical activity.

## Supplementary Materials

The following are available online at http://www.mdpi.com/2072-6643/11/1/148/s1, Table S1: The comparison of anthropometric measures, blood pressure, lipid profile, dietary intake, physical activity and leptin between men and women, Table S2: Linear regression analysis with the DII score as independent variable for men and women.
