# Supplementary material for: Dietary Inflammatory Index Score and Its Association with Body Weight, Blood Pressure, Lipid Profile, and Leptin in Indonesian Adults
Source: Nutrients. 2019 Jan 11;11(1):148. doi: 10.3390/nu11010148 (PMC6356884; doi:10.3390/nu11010148)
Supplement: Supplementary file 1 [file nutrients-11-00148-s001.pdf]

Supplementary Table 1. The comparison of anthropometric measures, blood pressure, lipid profile, dietary intake, physical activity and leptin between men and women

	**Men (n=252)**	**Women (n=251)**	**p**
Age (years)	41.1 ± 10.8	42.1 ± 9.6	0.500^
Anthropometric measures			
Body weight (kg)	65.4 ± 13.2	59.7 ± 12.3	<0.001^
Height (cm)	164.9 ± 6.4	152.0 ± 6.2	<0.001*
Body mass index (kg/m^2)	24.0 ± 4.4	25.9 ± 5.4	<0.001^
Body fat (%)	21.1 ± 6.0	33.9 ± 5.9	<0.001^
Waist circumference (cm)	84.4 ± 11.8	88.1 ± 13.4	0.003^
Hip circumference (cm)	91.4 ± 9.8	96.5 ± 11.9	<0.001^
Waist-to-hip ratio	0.92 ± 0.06	0.91 ± 0.07	0.094^
Blood pressure			
Systolic (mmHg)	131.0 ± 26.5	126.3 ± 23.7	0.020^
Diastolic (mmHg)	81.2 ± 15.8	78.2 ± 13.6	0.043^
Metabolic profile			
Triglycerides (mmol/L)	1.71 ± 0.91	1.42 ± 0.67	0.003^
HDL cholesterol (mmol/L)	1.07 ± 0.36	1.30 ± 0.54	<0.001^
Leptin (ng/mL)	2.7 ± 4.8	11.4 ± 9.6	<0.001^
Physical activity (METS-min/week)	6815 ± 6532	4747 ± 5071	<0.001^
Dietary intake			
Energy (kJ/day)	11 394 ± 4 621	10 277 ± 4 898	0.002^
Protein (energy%)	12.5 ± 3.3	12.6 ± 3.5	0.664^
Fat (energy%)	21.3 ± 9.8	25.0 ± 11.1	<0.001^
Carbohydrate (energy%)	66.1 ± 9.9	62.5 ± 11.4	<0.001^
Dietary Inflammatory Index score	1.07 ± 7.19	0.95 ± 7.4	0.898^
% overweight[a]	19.7	26.2	0.003[#]
% obese[b]	3.6	11.3	<0.001[#]

Values are presented as mean ± standard deviation, p value of difference between men and women, [a]body mass index ≥ 25 kg/m^2 or higher, [b]body mass index ≥ 30 kg/m^2 or higher, [a,b]percent of total study participants, * independent t-test, ^Mann-Whitney test, #Chi-square test

Supplementary Table 2. Linear regression analysis with the DII score as independent variable for men and women.

	**Men (n=252)**						**Women (n=251)**					
	**Model 0**		**Model I**		**Model II**		**Model 0**		**Model I**		**Model II**	
	**B**	***p***	**B**	***p***	**B**	***p***	**B**	***p***	**B**	***p***	**B**	***p***
Body weight (kg)	-0.157	0.180	-0.082[a]	0.194	-0.037[b]	0.643	0.121	0.249	0.062[a]	0.323	-0.020[b]	0.787
BMI (kg/m^2)	-0.049	0.204	-0.076[a]	0.224	-0.049[b]	0.535	0.025	0.593	0.022[a]	0.720	-0.084[b]	0.257
Body fat (%)	-0.040	0.446	-0.042[a]	0.487	-0.026[b]	0.731	0.023	0.650	0.015[a]	0.804	-0.075[b]	0.295
Waist circumference (cm)	-0.127	0.227	-0.071[a]	0.245	-0.011[b]	0.891	-0.031	0.789	-0.033[a]	0.578	-0.058[b]	0.414
Hip circumference (cm)	-0.082	0.345	-0.054[a]	0.379	-0.036[b]	0.638	0.107	0.294	0.053[a]	0.381	-0.039[b]	0.596
Systolic BP (mmHg)	0.173	0.460	0.049[a]	0.424	0.056[c]	0.440	0.261	0.196	0.066[a]	0.244	0.005[c]	0.938
Diastolic BP (mmHg)	0.069	0.622	0.034[a]	0.589	0.052[c]	0.485	0.181	0.120	0.086[a]	0.156	0.039[c]	0.574
Triglycerides (mmol/L)	-0.009	0.263	-0.067[a]	0.300	-0.059[c]	0.448	-0.0002	0.973	-0.008[a]	0.896	-0.044[c]	0.573
HDL cholesterol (mmol/L)	-0.005	0.160	-0.096[a]	0.136	-0.131[c]	0.102	0.006	0.230	0.084[a]	0.198	0.003[c]	0.971
Leptin (ng/mL)	0.055	0.281	0.081[a]	0.263	0.157[c]	0.019	0.160	0.070	0.120[a]	0.078	0.110[c]	0.089

Model 0, linear regression analysis without adjustment; Model I and Model II, linear regression analysis with adjustment; [a]linear regression analysis with adjustment for age; [b]linear regression analysis with adjusment for age, energy intake and physical activity; [c]linear regression analysis with adjustment for age, sex, body mass index, energy intake and physical activity. BP: blood pressure.
